# The ocular surface microbiome of rhesus macaques

**DOI:** 10.1186/s42523-025-00454-4

**Published:** 2025-08-20

**Authors:** Joelle K. Hass, Arthur G. Fernandes, Michael J. Montague, Armando Burgos-Rodriguez, Melween I. Martinez, Lauren J. N. Brent, Noah Snyder-Mackler, John Danias, Gadi Wollstein, James P. Higham, Amanda D. Melin

**Affiliations:** 1 Department of Anthropology and Archaeology, University of Calgary, Calgary, Alberta Canada; 2 Department of Neuroscience, University of Pennsylvania, Philadelphia, Pennsylvania USA; 3 University of Puerto Rico (Caribbean Primate Research Center), San Juan (Puerto Rico), USA; 4 University of Exeter (Center for Research in Animal Behavior), Exeter, UK; 5 School of Life Sciences, Arizona State University, Tempe, Arizona USA; 6 Center for Evolution and Medicine, Arizona State University, Tempe, Arizona USA; 7 Department of Ophthalmology, SUNY Downstate Health Sciences University, Brooklyn, New York USA; 8 Wills Eye Hospital, Philadelphia, Pennsylvania USA; 9 Vickie and Jack Farber Vision Research Center, Wills Eye Hospital, Philadelphia, Pennsylvania USA; 10 Department of Anthropology, New York University College of Arts & Science, New York, New York USA; 11 Department of Medical Genetics, University of Calgary, Calgary, Alberta Canada

**Keywords:** Eye microbiome, Nonhuman primates, MiSeq, 16S, Amplicon sequencing

## Abstract

**Background:**

The ocular surface microbiota (OSM) is important for eye health, and variations in OSM composition have been associated with multiple diseases in humans. Studies of OSM-disease dynamics in humans are confounded by lifestyle factors. Animal models provide a complementary approach to understanding biological systems, free from many confounds of human studies. Here, we provide the first study of the OSM of rhesus macaques, a premier animal model for eye health and disease. We describe the taxonomy of the rhesus macaque OSM, and explore compositional correlations with age, sex, and living condition.

**Methods:**

We analyzed eyelid and conjunctival microbiota swabs from 132 individual rhesus macaques (*Macaca mulatta*) (57 males, 75 females, 1–26 years old) from one captive and one free-ranging group using 16 S rRNA V3/V4 MiSeq sequencing. We investigated alpha diversity, beta diversity, and differential abundance.

**Results:**

We found several similarities between the top Phyla and Genera of the rhesus macaque OSM and those reported in human literature. Significantly higher alpha diversity, which may reflect age-related ocular surface mucous membrane integrity and immune function, was present in younger individuals compared to older ones. Higher alpha diversity was also present in free-ranging rhesus macaques compared to ones in captivity, possibly related to differences in diet, exercise, and medical exposures between macaques in different living conditions. Beta diversity was most strongly influenced by individual identity, followed by living conditions. Sex did not correlate with any OSM variation.

**Conclusions:**

In this study we describe the taxonomic composition of the rhesus macaque OSM, and identify significant differences in alpha and beta diversity according to individual nonhuman primate host variables and the surrounding environment. Our findings suggest composition of the nonhuman primate OSM is shaped by age-related physiology, individual identity, and external living conditions. Our results offer novel insights into an underexplored region of the primate microbiome and highlight the utility of rhesus macaques as a model system for investigating the links between the OSM, ocular health, and disease.

**Supplementary Information:**

The online version contains supplementary material available at 10.1186/s42523-025-00454-4.

## Background

The ocular surface microbiota (OSM) –the community of microorganisms living on the surface of the eye– plays an important role in regulating eye health, and OSM composition has been correlated with a number of diseases [1, 2]. The OSM was first described using traditional culture techniques in 1930 by Keilty [3]. However, research on the OSM has been slow to develop, largely due to its low biomass nature [4]. For example, the OSM was not included in the original Human Microbiome Project [5]. Despite several mechanisms which limit bacterial growth on the eye surface, including the mechanical clearance caused by the movement of the eyelids and the antibacterial nature of the tears, there is now a strong consensus that human eyes harbor a diverse community of microbiota [1, 2]. In recent years, studies using next generation sequencing techniques have shed light on the taxonomy of the OSM in healthy research participants [6–11], while other studies have begun to investigate how the microbiota may contribute to eye health through metabolic and immunologic mechanisms [12–14]. Correlations between OSM composition and a number of eye diseases have been identified, including dry eye disease, Sjogren syndrome, meibomian gland dysfunction, and glaucoma [12, 15–17]. Variation in OSM composition has also been correlated with several systemic conditions, such as diabetes and graft-versus-host disease [18–20]. Understanding the typical and pathological OSM is an important step for improving our knowledge of the pathogenesis of these diseases, and may shed light on both systemic and local interactions between the eye microbiota and the host.

Recent OSM studies in healthy human participants have revealed conflicting results on the relationship between demographic factors and OSM composition. For example, there are mixed results regarding the relationship between OSM diversity and age, with some studies showing higher diversity with increased age [9, 21], others showing lower diversity with age [22], and yet others showing no relationship at all [10, 23]. Similarly, evidence for sex differences has been reported [10, 21] or not [9] depending on the study. Individual identity, or inter-individual variation resulting from a complex combination of genetics, exposures, and experiences throughout an organism’s lifetime, has also been identified as an important factor in shaping the diversity of the OSM in humans [7]. Since OSM composition may be influenced by diverse individual host lifestyle factors including diet, exercise, use of medications and contact lenses, and overall host health [1, 2, 11], it is difficult to draw conclusions about age and sex dynamics with the OSM. Additionally, OSM studies have varied in methodology used to determine alpha and beta diversity, sequencing technologies, and filtering and decontamination parameters. Overall, there are extensive sociocultural and individual factors that contribute to variation in human eye microbiota composition, suggesting that the use of an appropriate animal model might prove to be highly valuable.

The study of non-human primates (NHPs) as model organisms for understanding health and aging processes complements human research and offers many advantages and insights. The rhesus macaque (*Macaca mulatta*) is a premier animal model for human health due to high similarity in DNA sequence (around 90%) and in anatomy and physiology [24]. With respect to the visual system, they demonstrate highly comparable eye anatomy, and experience similar naturally occurring visual decline as they age [24, 25]. Accordingly, they are valuable models for understanding myopia, cataracts, glaucoma, and macular degeneration [24–28]. Furthermore, recent studies extensively characterizing the microbiota on and in rhesus macaques have made valuable contributions to human health research [29]. However, there are currently no studies available on the rhesus macaque OSM either in healthy individuals, or as it relates to ocular disease.

We endeavor to provide a resource for the healthy rhesus macaque eye microbiome, and establish a foundational understanding of rhesus macaque OSM composition. In this study we (1) describe the taxonomy of the rhesus macaque OSM at two sampling sites commonly assessed in humans, the eyelid (EL) and the conjunctiva (CJ); and (2) examine OSM composition related to internal and external variables, including age and sex, and captive versus free-ranging environments. Since individual host characteristics and external environmental exposures can influence the relationships among microbiota and the host biological systems they interact with [30–32], we expected that OSM composition may vary with age, sex, and external living conditions. The results reported here will set the stage for future research on the rhesus macaque OSM as it relates to a variety of ocular diseases, and help to fill gaps of human OSM literature by describing OSM composition and relationships with host factors in an animal model with reduced variation in individual lifestyle, but still with high physiological and genetic relevance.

## Methods

### Study population

We studied 132 rhesus macaques from a colony managed by the Caribbean Primate Research Center (CPRC) through the University of Puerto Rico. 54 macaques (14 males, 40 females, 8–26 years old) were located in the Sabana Seca facility and 78 macaques (43 males, 35 females, 1–25 years old) were from the free-ranging population on the island of Cayo Santiago, Puerto Rico. The Cayo Santiago rhesus macaque population was established in 1938 with a group of 409 individuals imported from India [33], and the population now includes around 2000 individuals. A number of individuals have been transferred from Cayo Santiago to the Sabana Seca Field Station since 1984, where animals now live in captivity in outdoor corrals. The CPRC maintains a detailed census of both populations, recording the birth, death, and kinship information for all individuals; monkeys are recognized and tracked by tattoos, ear notches, and facial features. The CPRC provisions both of these primate populations with monkey chow and water. However, the free-ranging group on Cayo Santiago occasionally supplement their diet with native species of plants and invertebrates. The Cayo population also experiences natural weather conditions and individuals freely associate into, and move between, different social groups. Comparatively, the Sabana Seca individuals are housed in outdoor corrals, so while they experience some natural weather fluctuations, they have consistent shade and cover and individuals are not free to move between social groups. The Cayo population receives a tetanus inoculation at approximately one year of age, and certain individuals are trapped-and-released annually as part of longitudinal research studies [24]. The Sabana Seca group receives a tetanus inoculation every five years, an annual rabies vaccination, and ivermectin (an anthelmintic) as necessary. Additionally, this group receives tuberculin injections into their eyelid once every six months to monitor for tuberculosis infection. The Cayo group is not exposed to these further interventions.

### Sample collection/shipping/transportation

We collected eye microbiome swabs during animal exams that were scheduled as part of ongoing research studies. Prior to sample collection all monkeys were anesthetised by a trained CPRC veterinarian with intramuscular ketamine, and given 1–2 drops of topical anesthetic solution in each eye (tetracaine hydrochloride 0.5% or proparacaine hydrochloride 0.5% sterile ophthalmic solution).

We collected two types of samples using individual sterile swabs (*BBL CultureSwab*, Ottawa, ON): (1) EL swabs were collected from the lower eyelid margin by applying light friction and rubbing the swab from the medial to the lateral edge of the eyelid along the base of the eyelashes, avoiding contact with the conjunctiva; (2) CJ surface swabs were collected by rolling the swab back and forth 4–6 times along the bulbar conjunctival surface, while avoiding contact with the eyelid, eyelashes, or skin. Following sampling, we cut the swab tips into sterile 2mL cryogenic vials (*fisherscientific*, Pittsburgh, PA) for storage and transport. To minimize environmental contamination during the collection process, the researcher responsible for collection wore long sleeves, a hat, a face mask, and clean blue nitrile gloves. The scissors used for cutting the swab tips were sterilized with alcohol swabs between each sample. Additionally, sterile swabs were only opened at the exact time of sample collection, and the cryogenic vials for storage were only opened for the brief moment it took to place the swab tip inside, and immediately closed again. Swabs were placed on ice while in the field, and frozen at -80 °C within 5 h of collection.

Samples were transported from Puerto Rico to New York University (NYU) on dry ice and immediately stored at -80 °C upon arrival. DNA extractions were performed in a BSL-2 laboratory at NYU, and extracted DNA was shipped on dry ice to the University of Calgary for library preparation and sequencing.

### DNA extraction & sequencing

As a low biomass microbial niche, OSM samples are at high risk for contamination [4], and we followed strict protocols to minimize contamination during this study. DNA extractions were performed in a BioSafety Cabinet, with all equipment sterilized using 10% bleach, 70% ethanol, and 60 min of UV radiation prior to use. All disposable equipment was sterile, PCR-grade, DNA, and DNAse/RNAse free. The researcher performing the DNA extractions wore a sterile hair net, sleeve covers, face mask, and gloves throughout the procedure.

DNA extractions were performed using the Macherey-Nagel DNeasy-96-PowerSoil Pro kit (QIAGEN, Hilden, Germany) with a modified protocol. The CD2 inhibitor removal step was not performed, as per the manufacturer’s suggestion for low biomass samples. Negative controls were included in the form of extraction/reagent blanks: sample wells with sterile swabs were included in the extraction process as reagent controls, and processed along with the samples for all remaining steps.

We selected the sequencing parameters for this study based on recent human OSM studies in order to maximize the comparability of our results to current knowledge of the human OSM; many OSM studies have used Illumina MiSeq sequencing of the V3/V4 gene region [6, 8, 34]. Library preparation and sequencing were performed at the University of Calgary Centre for Health Genomics and Informatics (CHGI) core lab. Samples were processed into NGS libraries using Illumina 16 S Metagenomic Sequencing Library Preparation using an adjusted low biomass protocol. 11.5ul of stock sample was used as input into the first stage PCR. Adjustments to the library prep protocol to account for the low DNA input included: a reduction in PCR bead clean up elution volumes to 30ul for PCR clean up 1 and to 20ul for PCR clean up 2; adjustments to the indexing reaction include 3x volume increase in amplicon used along with a removal of PCR grade water. Libraries were sequenced on two MiSeq 600 cycle v3 sequencing runs, PE300 with 25% phiX spike in due to the low diversity nature of the libraries. Run 1 included an equal molar pool of 288 libraries and Run 2 included an equal molar pool of 258 libraries. Fifty-nine libraries were below the minimum concentration of 2ng/uL and were used ‘as is’. A total of 23,723,252 raw reads were produced with an average of 41,186 reads per sample for Run 1, and a total of 28,197,388 raw reads were produced with an average of 54,646 reads per sample for Run 2.

### Data analysis

We assessed the quality of the sequences using FastQC and MultiQC. We removed primers and adapters and performed quality trimming using Trimmomatic with the parameters fa:2:30:10:2:True LEADING:5 TRAILING:5 SLIDINGWINDOW:5:20 MINLEN:50. This means leading and trailing bases were trimmed immediately if they had a quality of 5 or less, and nucleotide bases were trimmed in a sliding window (5 bases long) if the average quality within that window fell below 20. Trimmed reads for Run 1 and Run 2 were then processed separately through the dada2 [35] pipeline in R 4.3.1, and the resulting taxonomy and sequence tables were merged for further downstream analysis. This resulted in 11,374,429 reads (average of 20,832 per sample), which clustered into 42,059 unique amplicon sequence variants (ASVs). Taxonomy was assigned using the SILVA v.138 database.

All downstream analyses were carried out in R using the packages *phyloseq*,* decontam*,* vegan*,* lme4*,* lmerTest*,* fantaxtic*,* microbiome*,* microbiomeMarker*,* ANCOMBC*, with visualizations using *ggplot2*,* ggpubr*, and *sjPlot*. We filtered the reads for contaminants using the *decontam* package at a threshold of 0.5. 627 ASVs were identified as contaminants based on the reagent blanks included in sequencing, and subsequently pruned from the dataset. We also removed ASVs uncharacterized at the Phyla level, chloroplasts and mitochondria, ASV singletons, and ASVs present in less than 2% of samples. Dong et al. (2022) [9] found that 85% of ASVs from the human OSM were present in less than 5% of samples. We chose 2% as a threshold to capture the diversity of this low biomass microbial community, where many ASVs might be expected to occur in less than 5% of samples but still represent meaningful biological taxa. After all quality control preprocessing steps, there were 1431 unique ASVs belonging to 7 Phyla and 63 Genera. Rarefaction (the process of randomly subsampling all samples down to a uniform sequencing depth) has been debated in the literature and critiqued for discarding meaningful biological information [36, 37], although recent work has combated this critique [38]. In OSM studies it is common to use alternative normalization strategies instead of rarefaction, especially due to the low biomass nature of this microbial niche [21, 34]. We have normalized our samples for read count as described below.

We measured alpha diversity using three standard metrics: the Shannon Diversity Index, Simpson Index, and Chao1 richness [39]. We visualized read count (number of reads per sample) against these metrics and identified a strong positive relationship for all three. To normalize the data for read count we plotted linear models with these metrics according to read count and extracted the residuals for use in all further analyses. We used Wilcoxon-signed rank tests to compare Shannon Index residuals, Simpson Index residuals, and Chao1 richness residuals between categorical groups of interest, including age (young versus old), sex (male versus female), living condition (captive versus free-ranging), sampled site (EL versus CJ), and eye laterality (left versus right). We also constructed linear mixed models to explore relationships between our variables of interest and alpha diversity variation; in all models age, sex, living condition, sequencing run, eye laterality, and sampled site were included as fixed effects with individual identity (represented by each macaque’s unique identification number) as a random effect. Including individual identity accounts for non-independent sampling, as each monkey was represented by multiple swabs (right and left eyes, EL and CJ). The inclusion of individual identity also allowed us to parse the influences of individual characteristics such as age and sex, versus the influence of overall host identity. To explore the most abundant taxa in the rhesus macaque OSM we transformed raw count data to relative abundances, and calculated the mean relative abundance for each taxon to identify the top most abundant Phyla and Genera. ‘Top Taxa’ were defined as the four most abundant Phyla and the ten most abundant Genera, which is a common practice in literature on the human OSM.

Current understandings of human ocular physiology suggest that older adults can experience lower ocular mucous membrane integrity, decreased tear production, and changes in immune function which may affect eye health [40]. Here we defined ‘old age’ as individuals in the top 75% quartile, which for this dataset was > 17.75 years of age (17.75–26.56 years old), and ‘younger’ monkeys -including young and middle-aged animals- (1-17.59 years old) below this threshold (see Supplementary Material 1 for all metadata). Based on life history traits, rhesus macaques are sometimes separated into further age groups containing pre-weaning individuals and pre-sexual maturity [41], but this dataset lacked sufficient numbers of these pre-adult individuals for a more granular comparison. Therefore, we have focussed our comparison on the effects of old age on microbiome composition.

We measured beta diversity using two metrics, Bray Curtis Dissimilarity and Aitchison Distance. We chose Bray Curtis due to its common use in the OSM literature to enable comparison of our results to existing studies, while Aitchison Distance was chosen due to recent papers recommending it for the highly compositional and sparse nature of microbiome datasets [42, 43]. We visualized structural relationships using Principal Coordinate Analysis (PCoA, Bray Curtis) and Principal Component Analysis (PCA, Aitchison Distance). We used PERMANOVA models to explore the significance of variables of interest in shaping beta diversity, including age, sex, individual identity, living condition, and sampled site, with eye laterality and sequencing run included to control for possible confounding effects.

We measured differential abundance (DA) using ANCOM-BC. ANCOM-BC is considered a conservative test and has been recommended for microbiome studies due to its ability to account for the compositional and sparse nature of microbiome datasets [44–46]. We filtered ANCOM-BC results to include Genera that were identified as differentially abundant according to the function (diff_ == TRUE), resistant to differential treatment of zeroes (ss_ == TRUE), and had a *q*-value of less than 0.05, with a log fold change of greater than |0.25|. This log fold change threshold was chosen for ANCOM-BC due to the highly conservative nature of the other filtering parameters. In addition, we provide alternative results using the DESeq2 test as supplemental material. DESeq2 is commonly used in microbiome literature, but was adapted from RNAseq purposes and has been criticized as less appropriate for the high sparsity nature of microbiome datasets [44, 46]. To enable comparison of our results with human literature, and to explore differences produced by analyzing the same dataset with different DA techniques, we provide the DESeq2 results in Supplementary Material 2.

## Results

After trimming and filtering, final analyses included samples from 131 individuals and a total of 251 eyes. The free-ranging group included samples from 77 individuals and 147 eyes (43 males, 34 females, 1–25 years old) and the captive group included samples from 54 individuals and 104 eyes (14 males, 40 females, ages 8–26 years old).

### The taxonomy of the rhesus macaque OSM

#### Top taxa combined

The final 1431 ASVs were assigned to 7 Phyla across the entire dataset of captive and free-ranging individuals, with the top 4 being *Firmicutes* (58.03 ± 17.5%), *Bacteroidota* (21.39 ± 14.9%), *Actinobacteriota* (14.23 ± 18.3%), and *Proteobacteria* (4.72 ± 7.8%). There were 63 total Genera, with the top 10 being *Prevotella_9* (12.05 ± 8.8%), *Streptococcus* (10.51 ± 10.7%), *Lactobacillus* (8.91 ± 7.19%), *Staphylococcus* (7.84 ± 14.8%), *HT002* (7.74 ± 5.44%), *Corynebacterium* (5.79 ± 12.2%), *Prevotella* (3.89 ± 4.55%), *Faecalibacterium* (3.70 ± 4.01%), *Ligilactobacillus* (3.17 ± 3.49%), and *Brevibacterium* (2.60 ± 5.9%). *Prevotella_9* is distinguished in the SILVA database from *Prevotella* as a genetically distinct sub-lineage within the greater *Prevotella* genus.

#### Top taxa in captive versus free-ranging

**Fig. 1:**
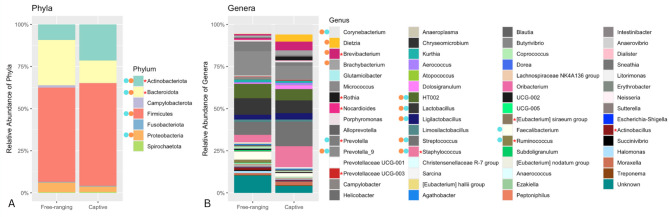
Taxa of the rhesus macaque ocular surface microbiota. Taxa denoted by an orange dot are Top Taxa in captive macaques, those with a blue dot are Top Taxa in free-ranging macaques. Taxa denoted with a red star were identified as differentially abundant between the captive and free-ranging groups using ANCOM-BC (*see Differential Abundance* section). (**A**) Relative abundance of phyla in free-ranging versus captive rhesus macaques. (**B**) Relative abundance of genera in free-ranging versus captive rhesus macaques

Within the captive group (*n* = 54) the top 4 Phyla were *Firmicutes* (60.94 ± 19.01%), *Actinobacteriota* (21.42 ± 20.70), *Bacteroidota* (13.41%±10.92%), and *Proteobacteria* (3.33 ± 7.03%); within the free-ranging group (*n* = 77) the top Phyla were *Firmicutes* (55.98 ± 15.97%), *Bacteroidota* (27.01 ± 14.75%), *Actinobacteriota* (9.16 ± 14.48%), and *Proteobacteria* (5.70 ± 8.19%)(Fig. 1A). The top ten Genera of the captive group were *Streptococcus* (14.55 ± 12.22%), *Staphylococcus* (12.47 ± 18.13%), *Prevotella_9* (8.35 ± 8.03%), *Lactobacillus* (7.55 ± 6.68%), *HT002* (6.75 ± 5.74%), *Corynebacterium* (6.05 ± 12.73%), *Brevibacterium* (5.18 ± 7.46%), *Dietzia* (4.11 ± 11.22%), *Ligilactobacillus* (3.77 ± 3.75%), and *Brachybacterium* (2.67 ± 4.49%); the top ten Genera of the free-ranging group were *Prevotella_9* (14.65 ± 8.39%), *Lactobacillus* (9.87 ± 7.39%), *HT002* (8.45 ± 5.11%), *Streptococcus* (7.66 ± 8.47%), *Corynebacterium* (5.61 ± 11.90%), *Prevotella* (5.31 ± 4.97%), *Faecalibacterium* (4.77 ± 4.29%), *Staphylococcus* (4.58 ± 10.88%), *Ligilactobacillus* (2.74 ± 3.22%), and *Ruminococcus* (1.91 ± 2.83%)(Fig. 1B).

### Alpha diversity & sampled site differential abundance

For metrics of alpha diversity, sampled site did not differ significantly in Shannon Index or Simpson Index. The average residual Chao1 richness of CJ samples (2.98 ± 26.50) was significantly higher than EL samples (-2.87 ± 36.63; Wilcoxon test, *p* = 0.0006, CI 95%= 2.492 to 8.499). Alpha diversity did not differ significantly between eye laterality by any metrics. Using ANCOM-BC to assess the DA of specific taxa, we identified that EL samples were enriched in *Peptoniphilus* compared to CJ samples, and CJ samples were comparatively enriched in *Dolosigranulum*.

### OSM composition related to age, sex, and living conditions

#### Alpha diversity

**Fig. 2:**
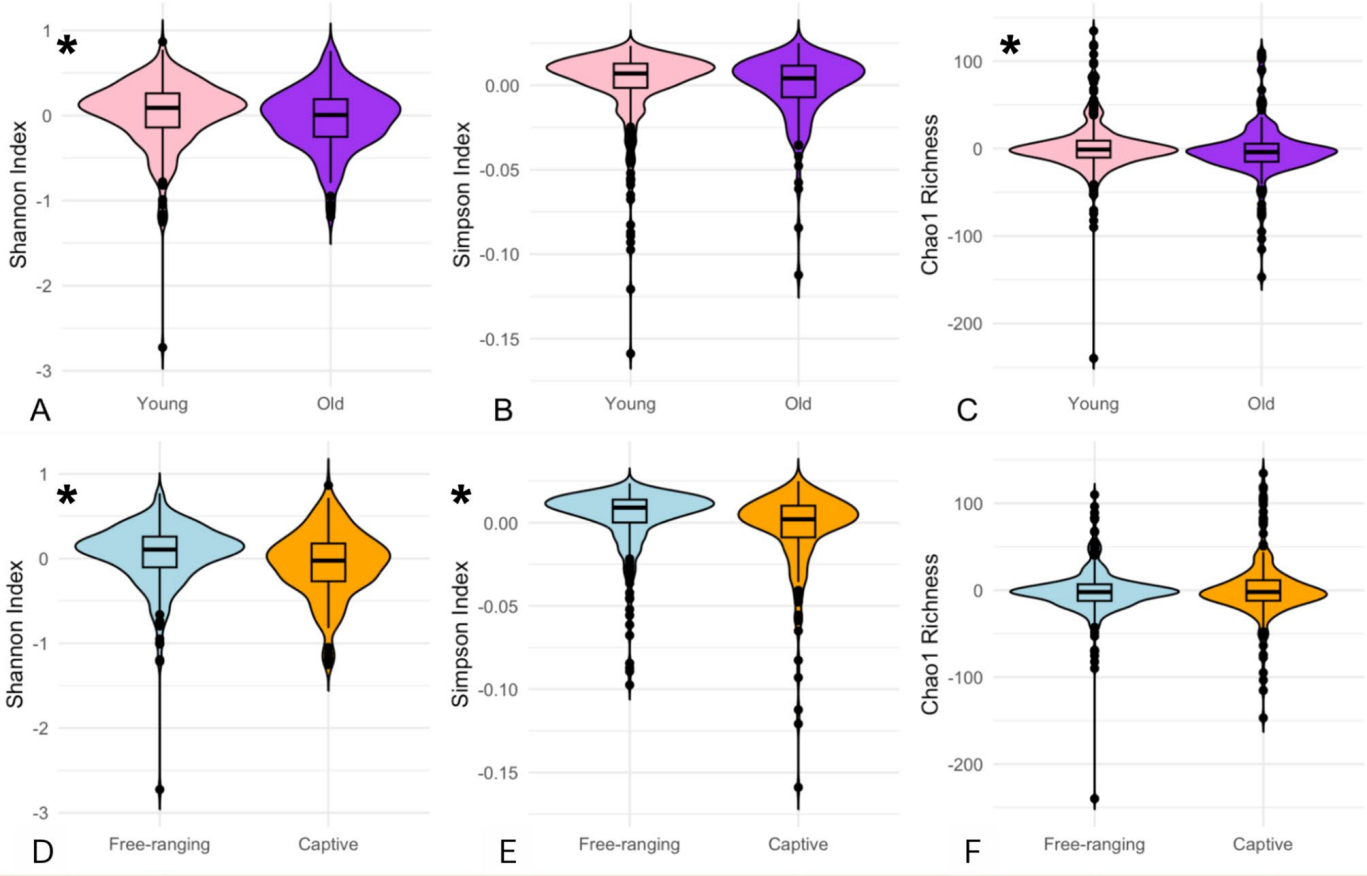
Measures of alpha diversity in young versus old and captive versus free-ranging rhesus macaques. *P*-value < 0.05 is denoted with an *. (**A**) Shannon Index (*p* = 0.013). (**B**) Simpson Index (*p* = 0.07398). (**C**) Chao1 richness (*p* = 0.034). (**D**) Shannon Index (*p* = 0.00020). (**E**) Simpson Index (*p* = 1.985e-06). (**F**) Chao1 richness (*p* = 0.7555)

The following results exploring associations between alpha diversity and the host variables of age, sex, and living condition did not differ when subset into CJ versus EL sampling locations, and we therefore combined all samples for our analyses. First, we directly compared alpha diversity metrics between the categorical groups of interest (age, sex, and living condition) using Wilcoxon-signed rank tests. The young group demonstrated significantly higher average residual Shannon Index (0.021 ± 0.394) and Chao1 Richness (1.860 ± 31.491) than the old group (Shannon Index: -0.041 ± 0.363, Wilcoxon test: *p* = 0.013, CI 95%= 0.017 to 0.144; Chao 1 Richness: -3.708 ± 33.252, Wilcoxon test: *p* = 0.034, CI 95%= 0.265 to 7.165), but showed no differences in Simpson Index (Fig. 2, A-C). Males and females did not differ significantly in any alpha diversity metrics. Free-ranging individuals demonstrated higher average residual Shannon Index (0.046 ± 0.370) and Simpson Index (0.003 ± 0.019) compared to the captive group (Shannon Index: -0.065 ± 0.396, Wilcoxon test: *p* = 0.00020, CI 95%= 0.058 to 0.179; Simpson Index: -0.004 ± 0.025, Wilcoxon test: *p* = 1.985e-06, CI 95%= 0.003 to 0.008), but Chao1 richness did not differ significantly (Fig. 2, D-F).

Next, we used linear modelling with the *lme4* package in R to explore the influence of age, sex, and living condition while controlling for individual identity in a combined model of alpha diversity variation. Because the data included both CJ and EL samples, we included sampled site as a variable in the models. In both Shannon Index and Simpson Index models, living condition was the only significant predictor of alpha diversity (*p* = 0.019 and *p* = 0.002 respectively; see Supplementary Material 3 for results tables), with captive individuals demonstrating lower alpha diversity by both metrics. In the Chao1 richness model none of the variables of interest (age, sex, and living condition) were significant predictors of richness. This suggests that living condition has the strongest influence on alpha diversity.

#### Beta diversity

In a combined PERMANOVA model using Bray Curtis Dissimilarity normalized through a relative abundance transformation, all variables came back as a statistically significant predictor of beta diversity: age (*p* = 0.007, R2 = 0.0027), sex (*p* = 0.012, R2 = 0.0024), living condition (*p* = 0.001, R2 = 0.0124), sampled site (*p* = 0.012, R2 = 0.0024), and individual identity (*p* = 0.001, R2 = 0.3291). However, only living condition and individual identity had R2 effect sizes demonstrating that they accounted for greater than 1% of variance in beta diversity, with living condition accounting for approximately 1.24% and individual identity for approximately 32.90%. The combined PERMANOVA model using Aitchison distance demonstrated similar results to the Bray Curtis model: age (*p* = 0.006, R2 = 0.0026), sex (*p* = 0.014, R2 = 0.0025), living condition (*p* = 0.001, R2 = 0.0133), sampled site (*p* = 0.025, R2 = 0.0024), and individual identity (*p* = 0.001, R2 = 0.3380). No clear clustering for the variables of interest based on either model was present in the PCoA or PCA visualizations. See Supplementary Material 4 for PERMANOVA results tables and visualization plots of beta diversity measures.

### Differential abundance

**Fig. 3:**
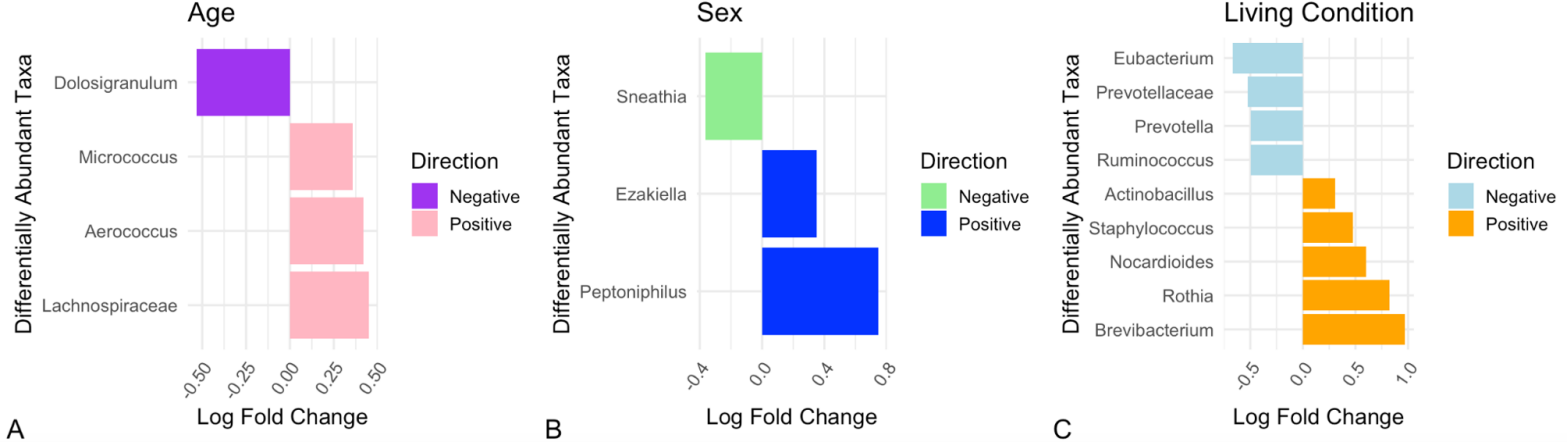
Differentially abundant microbial taxa identified using the statistical test ANCOM-BC. Effect size is represented by log fold change, where a value further from zero in either direction represents a stronger relationship. (**A**) Taxa identified as enriched in young rhesus macaques have a positive direction log fold change, whereas those identified as less abundant in young macaques have a negative log fold change. (**B**) Taxa identified as enriched in male rhesus macaques have a positive direction log fold change, whereas those identified as less abundant in male macaques have a negative log fold change. (**C**) Taxa identified as enriched in captive rhesus macaques have a positive direction log fold change, whereas those identified as less abundant in captive macaques have a negative log fold change

Using ANCOM-BC we identified that young macaques were enriched with the genera *Aerococcus*, *Micrococcus*, and *Lachnospiraceae NK4A136 group*, and less abundant in *Dolosigranulum* compared to older individuals (Fig. 3A). Regarding sex differences, we found that males were significantly enriched in *Ezakiella* and *Peptoniphilus*, and significantly lower in *Sneathia* (Fig. 3B). We also identified 5 genera as being significantly enriched in the captive group compared to the free-ranging group: *Actinobacillus*, *Staphylococcus*, *Nocardioides*, *Rothia*, and *Brevibacterium*; and 4 genera as being significantly lower in abundance in the captive group: *[Eubacterium] siraeum group*, *Prevotellaceae UCG-003*, *Prevotella*, and *Ruminococcus* (Fig. 3C).

## Discussion

In this study we aimed to identify the taxonomy of the rhesus macaque OSM and explore OSM composition as it relates to internal and external variables, including age, sex, and living condition. Overall, we identified 1431 ASVs spanning 7 phyla and 63 genera. We identified significantly different alpha diversity between older and younger rhesus macaques and between captive and free-ranging macaques, whereas sex did not correlate with alpha diversity by any measure. We also found that living condition and individual identity played a substantial role in shaping beta diversity, while age and sex did not. We identified specific taxa that differed in relative abundance between age groups, sex classes, and captive versus free-ranging populations.

### Taxonomy of the rhesus macaque OSM

The total number of unique microbial taxa reported in human OSM literature varies somewhat between studies. Patra et al. found a total of 3370 unique ASVs [47], Dong et al. reported 1731 OPUs (operational phylogenetic units) [9], and Ozkan et al. described 2465 zOTUs (zero radius operational taxonomic units) [11]; although it can be difficult to directly compare between different taxonomic clustering units such as ASVs, OPUs, and zOTUs [48], our result of 1431 ASVs appears similar to these human OSM findings. After trimming and filtering, we had a total of 11,374,429 reads, with an average of 20,832 reads per sample. Previous studies of the human OSM, having also used 16 S rRNA gene sequencing, have reported similar sequencing depths, including 6,017 reads per sample reported by Dong et al. [9], 13,169 by Ozkan et al. [cite 51], and 27,109 by Huang et al. [50]. Given that both our ASV count and our sequencing depth are similar to previous studies on the human OSM, this enables a discussion of similarities and differences between taxa reported in the human OSM literature and those we identified in the rhesus macaque OSM.

We identified *Firmicutes*, *Bacteroidota*, *Actinobacteriota*, and *Proteobacteria* as the top four most abundant phyla, accounting for over 98% of sequences. Across human studies, these same four phyla have been reported as the most abundant groups [49]. Additionally, we identified *Prevotella_9*, *Streptococcus*, *Lactobacillus*, *Staphylococcus*, *HT002*, *Corynebacterium*, *Prevotella*, *Faecalibacterium*, *Ligilactobacillus*, and *Brevibacterium* as the top ten most abundant genera, accounting for over 66% of sequences. Similar to our results, an amplicon sequencing study of the human OSM by Huang et al. described *Proteobacteria*, *Actinobacteria*, and *Firmicutes* as accounting for 96% of all sequencing reads, and their top ten most abundant genera accounted for 76% of reads [50]. Of particular interest, a metaanalysis of current human OSM literature by Delbeke et al. in 2021 concluded that the most common genera of the human OSM across studies were *Corynebacterium*, *Pseudomonas*, *Acinetobacter*, *Staphylococcus*, *Propionibacterium*, and *Streptococcus* [49]. Thus, *Corynebacterium*, *Staphylococcus*, and *Streptococcus* overlap with our results on the rhesus macaque OSM [49]. Despite numerous taxa that differed significantly between the captive and free-ranging groups, these three key genera were present in the Top Taxa of both populations. In a review by Peter et al. in 2023, the authors identified *Corynebacterium*, *Pseudomonas*, *Staphylococcus*, *Streptococcus*, *Acinetobacter*, *Propionibacterium*, *Bacillus*, *Agrobacterium*, *Sphingomonas*, *Cutibacterium*, and *Enhydrobacter* as the most abundant OSM genera, and *Chryseobacterium*, *Rothia*, *Massilia*, *Moraxella*, *Neisseria*, *Paracoccus*, *Ralstonia*, *Pedobacter*, and *Prevotella* as commonly identified but less abundant taxa [1]. Within this expanded list of common human OSM genera, we found *Rothia*, *Moraxella*, *Neisseria*, and *Prevotella* in the rhesus macaque OSM as well.

We also found a few key differences between the rhesus macaque OSM and the human OSM. Primarily, in humans, *Proteobacteria* is often identified as the top phylum by relative abundance [11, 50], whereas our results demonstrate *Firmicutes* as the most abundant phylum for rhesus macaques. For example, in 2016 Huang et al. found that *Proteobacteria* accounted for a mean of 46.50% of their sequencing reads, and *Firmicutes* only accounted for 15.50% [50]. In 2019, Ozkan et al. reported that a mean of 56.60% of reads were assigned to *Proteobacteria* and 23.80% belonged to *Firmicutes* [11]. Comparatively, we found that *Firmicutes* accounted for 58.03% of our total reads whereas *Proteobacteria* only accounted for 4.72%. Interestingly, we also found that a genus commonly identified across human OSM studies, *Pseudomonas*, was completely absent from the rhesus macaque OSM. *Pseudomonas* has been found embedded in human conjunctival tissue, suggesting it is a true member of the human OSM and not a transient environmental taxa [51]. *Pseudomonas* is a genus belonging to *Proteobacteria*, and thus its absence in the rhesus macaque OSM may partially account for the substantial difference in *Firmicutes*-to-*Proteobacteria* ratio in the rhesus macaque OSM compared to the human OSM. This may stand as an example of the influence of phylogeny on the OSM, as host evolutionary relationships can strongly shape microbial features [52].

### OSM composition related to age, sex, and living conditions

Older rhesus macaques demonstrated significantly lower alpha diversity compared to younger individuals, whereas sex did not correlate with any metric of alpha diversity. Differences in OSM composition with age may be associated with age-related changes in ocular surface mucous membrane integrity and immune function [40], which could influence microbiota growth on the ocular surface. Similar to our results, some human studies have found lower alpha diversity in older individuals [22], while others have found higher alpha diversity in aged groups [9, 21] or no effect of age on OSM alpha diversity [10, 23]. Beta diversity results also differ across the literature, with some studies similar to ours demonstrating little beta diversity variation with age [10, 23], and others suggesting the opposite [21, 22]. Regarding sex, some studies have found an influence of sex on alpha or beta diversity [10, 21], while others have not [11]. This variation may be attributed to a number of factors. First, the ocular surface is exposed to constant environmental fluctuations and perturbations, and therefore the OSM may be highly individual in nature. Indeed, we found that the variable accounting for the most variation in OSM composition was individual identity, which is inter-individual variation coming from a complex combination of genetics, exposures, and experiences throughout an organism’s lifetime. Our PERMANOVA results suggest that ASVs are much less likely to be shared between individuals; each monkey demonstrates a relatively unique OSM composition, which underscores how microbial community composition in a low biomass niche like the eye is heavily individualized. In support of our results, several human studies have also identified strong differences in OSM composition based on individual identity and discussed the apparent lack of a core human OSM [7, 51].

Notably, we found that young rhesus macaques were significantly enriched with *Micrococcus* and *Lachnospiraceae*. Certain members of these two genera are known producers of short chain fatty acids (SCFAs) and antioxidants such as reactive sulfur species [53, 54]. Studies on microbiota-retina relationships suggest that SCFAs and antioxidants may be important for regulating retinal function through anti-inflammatory and anti-oxidative pathways [55]. Changes in mucous membrane integrity and ocular surface immunity are common in aging primates, and may be related to this shift in OSM composition [40]. Altered ocular surface physiology could drive changes in OSM composition and vice versa, and thus future research investigating the potential influence of microbes locally at the surface of the eye is warranted.

When comparing free-ranging and captive rhesus macaque OSM composition, we found that numerous genera were differentially abundant between groups and the living condition variable was the second strongest predictor of variation in OSM composition in our PERMANOVA models. Our findings reflect patterns also noted in human studies which have found that the physical environment is a strong predictor of OSM composition [7, 56]. However, our results should also be considered in the context of the differing medical interventions experienced by the Sabana Seca and Cayo populations, particularly with regards to the tuberculin injections received by the captive group, which are injected into the eyelid every six months. These injections could temporarily affect tissue integrity and/or inflammation along the eyelid, potentially influencing OSM composition; however, tuberculin injections are not given concurrently, or close in time to our eye exams, limiting variation introduced by acute alterations in physiology at the ocular surface. Given that all captive individuals receive these injections, and no free-ranging individuals do, this variable cannot be separated from other aspects of the living condition, such as altered exposure to weather, social dynamics, diet, and human interactions. Overall, differences in microbiota composition between animals living in captive and free-ranging conditions are common. Two previous studies which investigated fecal microbiome composition between the Cayo and Sabana Seca macaque populations found that location [57] and housing site [58] (both equivalent to living condition) explained the greatest amount of variation in fecal microbiome composition. Interestingly, Compo et al. found that the Sabana Seca macaques demonstrated higher microbial diversity and richness compared to the free-ranging group [58], suggesting the OSM as a low biomass niche may respond differently to environmental factors than the high biomass gastrointestinal niche.

### OSM composition relevant to ocular physiology

Two genera identified in the Top Taxa of both captive and free-ranging rhesus macaques may be of particular interest for their influence on ocular physiology. In humans, *Corynebacterium* is known for its role in regulating local immune responses on mucous membranes, and specifically for upregulating the antimicrobial action of tears on the ocular surface [13]. Its shared presence in the OSM of rhesus macaques may suggest it plays a similar role, and warrants further investigation. *Staphylococcus* is also a common member of human skin and mucous membranes, and despite its presence in the human OSM it is associated with several types of eye infections [59]. Its presence in the rhesus macaque OSM offers supporting evidence that *Staphylococcus* is part of a healthy OSM composition, and its pathogenicity may depend on other factors. Overall, the use of sequencing techniques that can identify species- and strain-level taxonomic composition of the OSM may clarify the role of specific subgroups within genera of interest. Future studies using metagenomic, metabonomic, and metaproteomic approaches are needed to investigate the potential functional roles of microbial taxa on the ocular surface.

### Limitations and future directions

Our study is limited by several factors. Illumina MiSeq sequencing does not offer insight into strain-level variation or to non-bacterial microbiota members, and cannot shed light on the functional gene pathways expressed by the present organisms. We hope our study will prompt future investigation of the NHP OSM using deeper and more specific sequencing and other molecular techniques, such as metagenomics, metabonomics, and metaproteomics, to enable more detailed and functional insight. Although we followed strict contamination-avoidance protocols, we did not collect environmental controls, which may have prevented us from removing some environmentally-introduced ASVs during analyses. Future studies that include environmental controls will overcome this possible limitation. However, despite these limitations, our study demonstrates that low-biomass OSM samples can be successfully collected and sequenced from NHPs, and we provide first characterization of rhesus macaque OSM as well as demonstrate intriguing age-related variation that can be explored in more depth in future studies.

## Conclusions

We provide novel insight into the rhesus macaque ocular surface microbiome, reveal variation predicted by age and living condition, and offer intriguing insights to an unexplored biogeographical microbial niche in NHPs. Intriguingly, we find that rhesus macaques share the same top four most abundant phyla as those reported in the human OSM, along with several shared genera. However, there also appear to be key differences between rhesus macaque and human OSM composition, suggesting the potential influence of phylogeny on shaping primate OSM composition. Furthermore, we found four genera that differed between young and old rhesus macaques, several of which may be associated with age-related changes in immunity and membrane integrity at the ocular surface. Additionally, several of the top most abundant genera shared between captive and free-ranging groups may be capable of influencing ocular physiology, and warrant further investigation using more sensitive sequencing technologies to explore their functional capabilities. Our results extend the field of OSM research to NHPs and provide an introduction to the rhesus macaque OSM in particular. These results enhance the value of rhesus macaques as a translational model, may offer a promising avenue for improving medical models of OSM-related diseases, and expand our understanding of the basic biology and physiology of host-OSM relationships.

## Supplementary Information

Below is the link to the electronic supplementary material.

Supplementary Material 1

Supplementary Material 2

Supplementary Material 3

Supplementary Material 4

Supplementary Material 5

## Data Availability

The code for this study is available in Supplementary Material 5. The datasets generated and/or analysed during the current study are available in the NIH SRA repository, [https://www.ncbi.nlm.nih.gov/bioproject/PRJNA1221637].

## Abbreviations

ASV: Amplicon sequence variant
CJ: Conjunctiva
CPRC: Caribbean primate research center
DA: Differential abundance
EL: Eyelid
NHP: Nonhuman primate
OSM: Ocular surface microbiome
PCA: Principal component analysis
PCoA: Principal coordinate analysis
