# Supplementary material for: The ocular surface microbiome of rhesus macaques
Source: Anim Microbiome. 2025 Aug 20;7:88. doi: 10.1186/s42523-025-00454-4 (PMC12366034; doi:10.1186/s42523-025-00454-4)
Supplement: Supplementary file 2 — Supplementary Material 2 [file 42523_2025_454_MOESM2_ESM.docx]

**Alternative Differential Abundance Results (DESeq2)**

DESeq2 has been commonly used in microbiome literature and identifies specific ASVs that differ between groups of interest. The parameters for inclusion of DESeq2 results for this supplementary comparison included: a log2 fold change greater than |2.5| and an adjusted p-value less than 0.05. In two papers which assessed the efficacy of various microbiome differential abundance analysis techniques DESeq2 and ANCOM-BC performed well on different types of datasets, and both papers recommend reporting multiple differential abundance metrics per dataset [44, 46]. Disagreement in results may come from the different treatment of zeroes by the DESeq2 and ANCOM-BC functions, which can have a significant impact in datasets with high sparsity.

We identified that conjunctival samples were significantly enriched in one ASV from the genus *Kurthia* (ASV 152) compared to eyelid samples (Table 1).

Table 1

| Genus | ASV(s) | Enrichment Group | Overlap with ANCOM-BC |
| --- | --- | --- | --- |
| *Kurthia* | 152 | Conjunctiva | Not identified |

We also identified one ASV from the genus *Prevotella* (ASV 137) as being enriched in young macaques compared to old macaques, and 5 ASVs belonging to two genera as being less abundant in young macaques: *Dolosigranulum* (ASVs 482, 459, 389, & 396) and *Moraxella* (ASV 269)(Table 2).

Table 2

| Genus | ASV(s) | Enrichment Group | Overlap with ANCOM-BC |
| --- | --- | --- | --- |
| *Prevotella* | 137 | Young | Not identified |
| *Dolosigranulum* | 482, 459, 389, & 396 | Old | Agreement |
| *Moraxella* | 269 | Old | Not identified |

In the sex comparison we identified that males have a significantly lower abundance of 2 ASVs from the genus *Corynebacterium* (ASVs 82 & 109)(Table 3).

Table 3

| Genus | ASV(s) | Enrichment Group | Overlap with ANCOM-BC |
| --- | --- | --- | --- |
| *Corynebacterium* | 82 & 109 | Female | Not identified |

For living condition we identified 31 ASVs belonging to 8 genera that were enriched in the captive macaques compared to the free-ranging group: *Brachybacterium* (ASVs 188 & 645), *Brevibacterium* (ASVs 190, 113, 177, & 198), *Dietzia* (ASV 123), *Ligilactobacillus* (ASVs 385, 420, 554, 384, 195, 492, 502, & 751), *Prevotella_9* (ASVs 238, 263, 362, 372, 470, & 368), *Prevotellaceae UCG-003* (ASV 483), *Rothia* (ASVs 497, 267, 562, 673, & 586), and *Staphylococcus* (ASVs 370, 487, 250, & 699). We also identified 37 ASVs from 7 genera as being significantly less abundant in the captive group compared to the free-ranging: *Campylobacter* (ASVs 322 & 344), *Faecalibacterium* (ASV 31), *Halomonas* (628 & 169), *Prevotella_9* (ASVs 740, 580, 383, 454, 709, 553, 313, 410, 275, 291, 523, 324, 345, 244, 373, 220, 355, 376, 287, 339, 490, 215, 337, 326, 254, 209, 219, & 205), *Ruminococcus* (ASVs 300 & 151), *Sneathia* (ASV 681), and *UCG-005* (ASV 432)(Table 4).

Table 4

| Genus | ASV(s) | Enrichment Group | Overlap with ANCOM-BC |
| --- | --- | --- | --- |
| *Brachybacterium* | 188 & 645 | Captive | Not identified |
| *Brevibacterium* | 190, 113, 177, & 198 | Captive | Agreement |
| *Dietzia* | 123 | Captive | Not identified |
| *Ligilactobacillus* | 385, 420, 554, 384, 195, 492, 502, & 751 | Captive | Not identified |
| *Prevotella_9* | 238, 263, 362, 372, 470, & 368 | Captive | Not identified |
| *Prevotellaceae UCG-003* | 483 | Captive | Disagreement |
| *Rothia* | 497, 267, 562, 673, & 586 | Captive | Agreement |
| *Staphylococcus* | 370, 487, 250, & 699 | Captive | Agreement |
| *Campylobacter* | 322 & 344 | Free-ranging | Not identified |
| *Faecalibacterium* | 31 | Free-ranging | Not identified |
| *Halomonas* | 628 & 169 | Free-ranging | Not identified |
| *Prevotella_9* | 740, 580, 383, 454, 709, 553, 313, 410, 275, 291, 523, 324, 345, 244, 373, 220, 355, 376, 287, 339, 490, 215, 337, 326, 254, 209, 219, & 205 | Free-ranging | Not identified |
| *Ruminococcus* | 300 & 151 | Free-ranging | Agreement |
| *Sneathia* | 681 | Free-ranging | Not identified |
| *UCG-005* | 432 | Free-ranging | Not identified |
