# Supplementary material for: The ocular surface microbiome of rhesus macaques
Source: Anim Microbiome. 2025 Aug 20;7:88. doi: 10.1186/s42523-025-00454-4 (PMC12366034; doi:10.1186/s42523-025-00454-4)
Supplement: Supplementary file 3 — Supplementary Material 3 [file 42523_2025_454_MOESM3_ESM.docx]

**Results for linear mixed effects models of Shannon and Simpson alpha diversity**

| **Fixed Effect** | **Estimate** | **Std. Error** | **df** | **t value** | **Pr(>\|t\|)** |
| --- | --- | --- | --- | --- | --- |
| (Intercept) | 0.051 | 0.068 | 242.62 | 0.76 | 0.450 |
| SITESSFS | -0.111 | 0.047 | 123.91 | -2.38 | 0.019* |
| OldvsYoungYoung | 0.058 | 0.046 | 119.72 | 1.26 | 0.211 |
| SEXM | 0.005 | 0.046 | 121.48 | 0.11 | 0.914 |
| RunRun2 | -0.017 | 0.047 | 404.59 | -0.35 | 0.724 |
| ConjunOrEyelidEyelid | -0.035 | 0.031 | 351.81 | -1.11 | 0.266 |
| RightOrLeftRight | -0.040 | 0.042 | 463.40 | -0.94 | 0.346 |

**Table 1.** Results for linear mixed effects model of residual Shannon Index. P-value <0.05 denoted with an *.

| **Fixed Effect** | **Estimate** | **Std. Error** | **df** | **t value** | **Pr(>\|t\|)** |
| --- | --- | --- | --- | --- | --- |
| (Intercept) | 6.884e-03 | 3.791e-03 | 246.6 | 1.816 | 0.071 |
| SITESSFS | -8.287e-03 | 2.570e-03 | 125.9 | -3.224 | 0.002 ** |
| OldvsYoungYoung | 8.382e-04 | 2.547e-03 | 121.6 | 0.329 | 0.743 |
| SEXM | -1.085e-03 | 2.523e-03 | 123.5 | -0.430 | 0.668 |
| RunRun2 | -4.024e-03 | 2.669e-03 | 391.4 | -1.507 | 0.133 |
| ConjunOrEyelidEyelid | -5.805e-04 | 1.799e-03 | 354.7 | -0.323 | 0.747 |
| RightOrLeftRight | -2.678e-03 | 2.406e-03 | 465.4 | -1.113 | 0.266 |

**Table 2.** Results for linear mixed effects model of residual Simpson Index. P-value <0.05 denoted with an *.
