# Supplementary material for: The ocular surface microbiome of rhesus macaques
Source: Anim Microbiome. 2025 Aug 20;7:88. doi: 10.1186/s42523-025-00454-4 (PMC12366034; doi:10.1186/s42523-025-00454-4)
Supplement: Supplementary file 4 — Supplementary Material 4 [file 42523_2025_454_MOESM4_ESM.docx]

**Results for PERMANOVA models of Bray-Curtis and Aitchison beta diversity**

| **Term** | **Df** | **SumOfSqs** | **R²** | **F** | **Pr(>F)** | **Significance** |
| --- | --- | --- | --- | --- | --- | --- |
| SITE | 1 | 2.761 | 0.012 | 6.676 | 0.001 | *** |
| SEX | 1 | 0.541 | 0.002 | 1.307 | 0.012 | * |
| OldvsYoung | 1 | 0.601 | 0.003 | 1.455 | 0.007 | ** |
| RightOrLeft | 1 | 0.787 | 0.004 | 1.904 | 0.001 | *** |
| ConjunOrEyelid | 1 | 0.541 | 0.002 | 1.309 | 0.012 | * |
| Run | 1 | 1.328 | 0.006 | 3.211 | 0.001 | *** |
| ID | 127 | 73.185 | 0.329 | 1.394 | 0.001 | *** |
| Residual | 345 | 142.666 | 0.641 |  |  |  |
| **Total** | 478 | 222.411 | 1.000 |  |  |  |

**Table 1.** Bray-Curtis PERMANOVA results. Fixed effects accounting for >1% of variance (R2 > 0.01) denoted in red.

| **Term** | **Df** | **SumOfSqs** | **R²** | **F** | **Pr(>F)** | **Significance** |
| --- | --- | --- | --- | --- | --- | --- |
| SITE | 1 | 15713 | 0.013 | 7.280 | 0.001 | *** |
| SEX | 1 | 2966 | 0.003 | 1.374 | 0.014 | * |
| OldvsYoung | 1 | 3023 | 0.003 | 1.401 | 0.006 | ** |
| RightOrLeft | 1 | 4869 | 0.004 | 2.256 | 0.001 | *** |
| ConjunOrEyelid | 1 | 2782 | 0.002 | 1.289 | 0.025 | * |
| Run | 1 | 10059 | 0.008 | 4.660 | 0.001 | *** |
| ID | 127 | 400404 | 0.338 | 1.461 | 0.001 | *** |
| Residual | 345 | 744671 | 0.629 |  |  |  |
| **Total** | 478 | 1184488 | 1.000 |  |  |  |

**Table 2.** Aitchison PERMANOVA results. Fixed effects accounting for >1% of variance (R2 > 0.01) denoted in red.


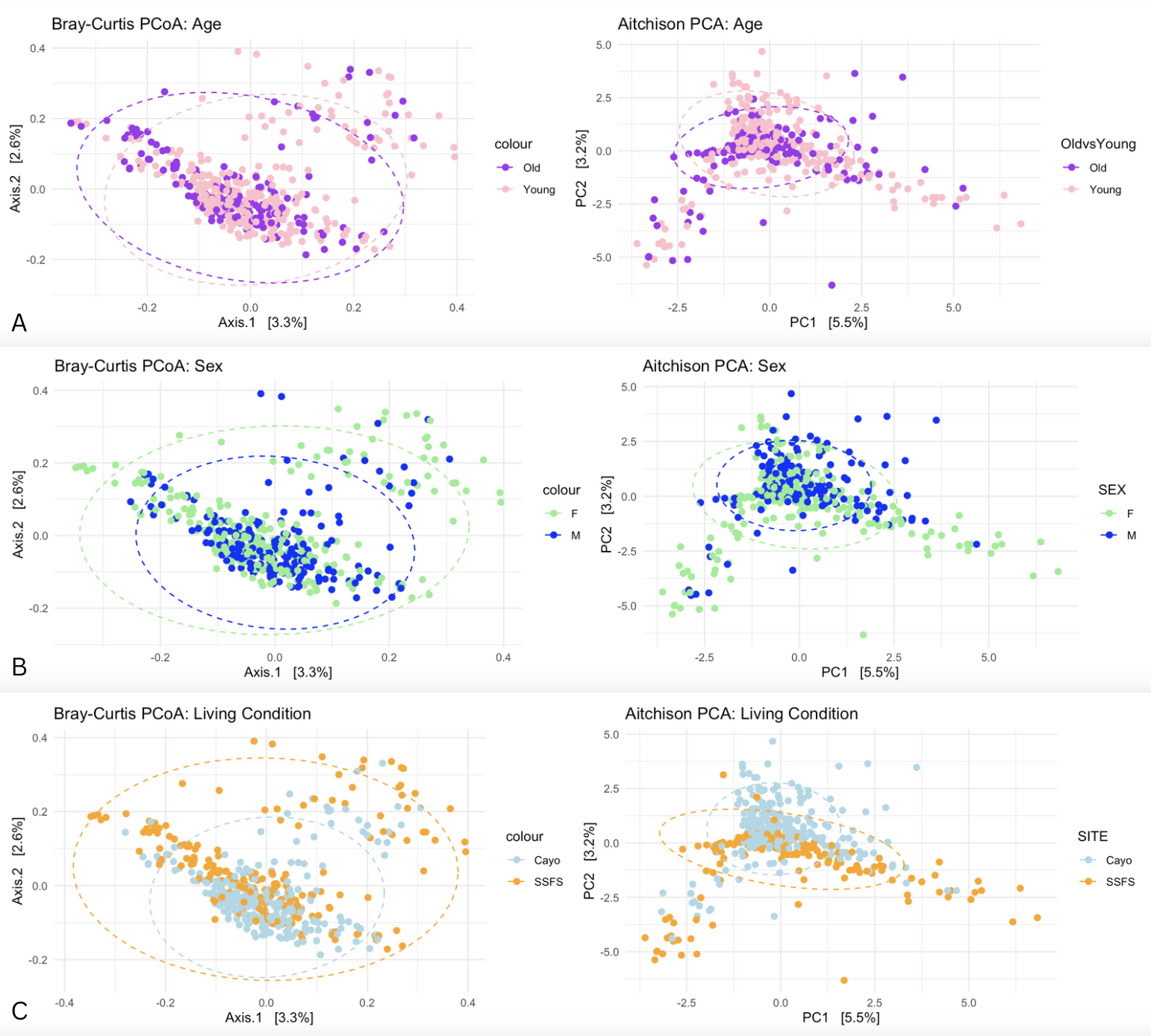


**Figure 1.** Visualization plots for beta diversity statistics. A. Bray-Curtis Dissimilarity PCoA and Aitchison Distance PCA plots of young versus old rhesus macaques. B. Bray-Curtis Dissimilarity PCoA and Aitchison Distance PCA plots of male versus female rhesus macaques. C. Bray-Curtis Dissimilarity PCoA and Aitchison Distance PCA plots of captive versus free-ranging rhesus macaques.
